# Fundamental Questions in Biology

**DOI:** 10.1371/journal.pbio.0040300

**Published:** 2006-09-12

**Authors:** Simon A Levin

## Abstract

The pace of our understanding of biology has engendered increasing specialization but there are still common fundamental challenges that unify biology and should form the core of future research.

Not so long ago, virtually every major university had a department of biology, or perhaps bookend departments of zoology and botany, which complemented physics, chemistry, mathematics, and possibly geology to form its science foundation. Biology was, at least compared to the field today, an integrated discipline, from the molecular and cellular to the ecosystem, firmly resting on Darwinian principles. Weekly colloquia drew biologists from across the spectrum, whether the topic was the genetic code, the nature of the synapse, or the Cambrian Radiation.

But biology has seen its own radiation and is just starting to catch up with this explosion. The amazing pace of advance in our understanding of biology has, perhaps unavoidably, engendered increasing specialization. Much of that advancement has involved the development of new tools, both in the laboratory and in computer models, and this has been dependent on the migration into biology departments of tools and people from physics, mathematics, chemistry, and elsewhere. These new collaborators have catalyzed rapid progress on specific problems, but they often have little interest in the broader scope of biology. Even traditional biologists with broader interests may not have the time to indulge outside of their own research areas because of the speed of scientific progress in those areas and the competitive nature of contemporary science. Departments of biology or botany/zoology have split and split again, producing departments of cell and molecular biology, ecology and evolutionary biology, neurobiology and behavior, genetics and development, physiology, and so on, reflecting the particular cultures of the specific institutions. Where departments of biology have remained intact, intradepartmental asymmetries in quality or funding potential have created tensions and siege mentalities and have encouraged university administrators to follow the money and to accept the fallacious argument that areas that require or attract less funding are hence outdated and dispensable.

But the situation may be changing. The rapid accumulation of information from genomics has reached a point where attention must be turned, if it has not already, to what the now vast library of genetic information means for how organisms function in their natural environments, and indeed for how ecological communities operate. Metagenomic methods are being applied to the collection of storehouses of genetic information about whole ecosystems, especially the oceans; but such information is of limited value unless one understands how that information is organized, how it is distributed over the biota, and why specific genes are associated with particular regions of the ecosystem. Are there particular conditions that select for novelty and for high mutation or recombination rates? What about for cooperative behavior? What is the relationship between the distribution of specific viral genes and the genes of other organisms, and can we begin to infer from this distributional information the possible role of viruses in mediating oceanic diversity?

At the core of this potential future shift in biological sciences is the recognition that all biological systems are what have come to be known as complex adaptive systems, in which macroscopic patterns reflect the collective dynamics of individual units at lower levels of organization and feed back to affect those more microscopic dynamics. Evolutionary changes operate on multiple levels and multiple scales: from cells, to organisms, to populations, to communities and the biosphere. As my Princeton colleague, Philip Anderson, wrote years ago, “more is different.” Although the details at lower levels govern the behavior at higher levels, understanding those details is not sufficient for understanding how macroscopic patterns emerge or how natural selection operates at lower levels to lead to those patterns. Where those patterns refer to properties of the organism, natural selection operates to modify the details, such as the rules that govern organismal development due to feedbacks from fitness differences among organisms. On the other hand, where those properties refer to those of the biosphere, there is no comparable process of natural selection choosing among competing biospheres. What properties arise are hence largely emergent, reflecting selective events at much lower levels of organization. This is the principal reason that our biosphere is in trouble. It also emphasizes the importance of understanding at what levels selection operates most strongly.

The questions that biologists from diverse subdisciplines are asking have commonalities that make clear the continued existence of fundamental challenges that unify biology and that should form the core of much research in the decades to come. Some of these questions are as follows: What features convey robustness to systems? How different should we expect the robustness of different systems to be, depending on whether selection is operating primarily on the whole system or on its parts? How does robustness trade off against adaptability? How does natural selection deal with environmental noise and the consequent uncertainty at diverse scales? When does synchrony emerge, and what are its implications for robustness? When and how does cooperative behavior emerge, and can we derive lessons from evolutionary history to foster cooperation in a global commons?

These are among what we identify as fundamental questions in biology, cutting across subdisciplines and with the potential to reunify the subject. To encourage recognition of these challenges, *PLoS Biology* is publishing a series of brief discussion papers raising core issues and designed to be provocative (the first in the series is published today [DOI: 10.1371/journal.pbio.0040299]). Contributions to the Challenges Series are encouraged; ideas should be sent to biology_editors@plos.org.

